# Improving eye care in residential aged care facilities using the Residential Ocular Care (ROC) model: study protocol for a multicentered, prospective, customized, and cluster randomized controlled trial in Australia

**DOI:** 10.1186/s13063-018-3025-5

**Published:** 2018-11-26

**Authors:** Edith E. Holloway, Marios Constantinou, Jing Xie, Eva K. Fenwick, Eric A. Finkelstein, Ryan E. K. Man, Michael Coote, Jonathan Jackson, Gwyn Rees, Ecosse L. Lamoureux

**Affiliations:** 1 Centre for Eye Research Australia, Royal Victorian Eye and Ear Hospital, Level 7, 32 Gisborne Street, East Melbourne, VIC 3002 Australia; 2 Ophthalmology, Department of Surgery, University of Melbourne, Grattan Street, Parkville, VIC 3010 Australia; 3 Singapore Eye Research Institute, Singapore National Eye Centre, Singapore, Singapore; 4 Duke-National University of Singapore Medical School, 8 College Road Level 4, Singapore, 169857 Singapore; 5 Department of Ophthalmology, Royal Victoria Hospital, Belfast Health & Social Care Trust, 274 Grosvenor Road, Belfast, BT12 6BA Northern Ireland, UK; 6 Australian College of Optometry, Corner Keppel and Cardigan Street, Carlton, VIC 3053 Australia

**Keywords:** Vision impairment, Residential care, Eye care model, Protocol, Trial

## Abstract

**Background:**

Older adults in residential aged care facilities have unnecessarily high levels of vision impairment (VI) which are largely treatable or correctable. However, no current comprehensive eye health service model exists in this setting in Australia. We aimed to determine the clinical, person-centered, and economic effectiveness of a novel eye care model, the Residential Ocular Care (ROC).

**Methods/design:**

This protocol describes a multicentered, prospective, randomized controlled trial. A total of 395 participants with distance vision < 6/12 (0.30 LogMAR) and/or near vision N8 (1.00 M) or worse will be recruited from 38 urban and rural aged care facilities across Victoria, Australia. Aged care facilities will be randomized (1:1) to one of two parallel groups. Participants in the ROC group will receive a comprehensive and tailored eye care pathway that includes, as necessary, refraction and spectacle provision, cataract surgery, low vision rehabilitation, and/or a referral to an ophthalmologist for funded treatment. Usual care participants will be referred for an evaluation to the eye care service associated with the facility or an eye care provider of their choice. The primary outcome will be presenting near and distance vision assessed at the two- and six-month follow-up visits, post baseline. Secondary outcomes will include vision-specific quality of life, mobility, falls, depression, and eye care utilization at two and six months. An incremental cost-effectiveness analysis will also be undertaken.

**Discussion:**

The ROC study is the first multicentered, prospective, customized, and cluster randomized controlled trial in Australia to determine the effectiveness of a comprehensive and tailored eye care model for people residing in aged care facilities. Results from this trial will assist health and social care planners in implementing similar innovative models of care for this growing segment of the population in Australia and elsewhere.

**Trial registration:**

Australian and New Zealand Clinical Trials Registry, ACTRN12615000587505. Registered on 4 June 2015 – retrospectively registered.

**Electronic supplementary material:**

The online version of this article (10.1186/s13063-018-3025-5) contains supplementary material, which is available to authorized users.

## Background

Globally, around 223 million people have vision impairment (VI) or blindness [1]. VI will continue to be a major contributor to the global burden of disease as a result of the rapidly aging population, as this segment of the population has the highest incidence of ocular conditions [2]. Aging leads to a decline in visual acuity (VA), a measure of the eye’s ability to resolve fine detail, resulting from a deterioration of the visual function system. VI is a significant problem in older adults as it increases disability and the risk of falls [3–5] and reduces vision-specific functioning, emotional health, independence, and participation in activities of daily living [5, 6]. The prevalence of VI in aged care facilities is reported to be significantly greater than in community-dwelling older adults [7–9]. Up to 60% of aged care residents have VI [5], compared to 5% of older adults aged > 70 years living independently in the community [10].

The prevalence of VI in aged care settings remains high due to limited access to eye care services [11]. This is despite the major causes of VI in older adults, such as uncorrected (and under-corrected) refractive error and cataracts, being correctable or treatable [5, 11, 12]. Adequate refractive correction, for example, could improve vision in up to 50% of individuals in nursing home residents [13]. Furthermore, prospective studies have shown that residents who receive correction [11] or cataract surgery [13] demonstrate short-term improvements (two and four months after intervention) in vision, quality of life (QoL) and increased participation in activities of daily living. Benefits have also been reported for mental health outcomes, with fewer depressive symptoms and lower levels of distress found in residents following active treatment compared to those with no correction or surgery [11].

For ocular conditions that cause irreversible or progressive vision loss, such as age-related macular degeneration (AMD), glaucoma, and diabetic retinopathy (DR), remaining vision can still be maximized through the provision of low vision rehabilitation. Few well-designed studies have examined the impact of low vision rehabilitation in residential care settings. Longitudinal studies, however, in community-living older adults have found improvements in vision-related QoL and participation in daily activities following prescription of low vision devices [14, 15].

To date, no study has evaluated the impact of providing comprehensive eye care services to individuals with a broad spectrum of ophthalmic conditions and residing in aged care facilities. Therefore, we will implement a prospective, customized, multicentered, and cluster randomized controlled trial (RCT) to evaluate the clinical, person-centered, and cost-effectiveness of a new model of eye care. We will compare the effectiveness of Residential Ocular Care (ROC) with usual care on improving presenting and best corrected near and distance vision (primary outcome) at the two- and six-month follow-up visits. Secondary outcomes will assess the effectiveness of ROC on improving vision-specific QoL, daily functioning, health-related QoL, depressive symptoms, rate of falls, and eye care utilization, compared to the usual care group at the two- and six-month visits, after baseline, and quantify the cost-effectiveness of ROC at six months after baseline.

## Methods and design

**Fig. 1:**
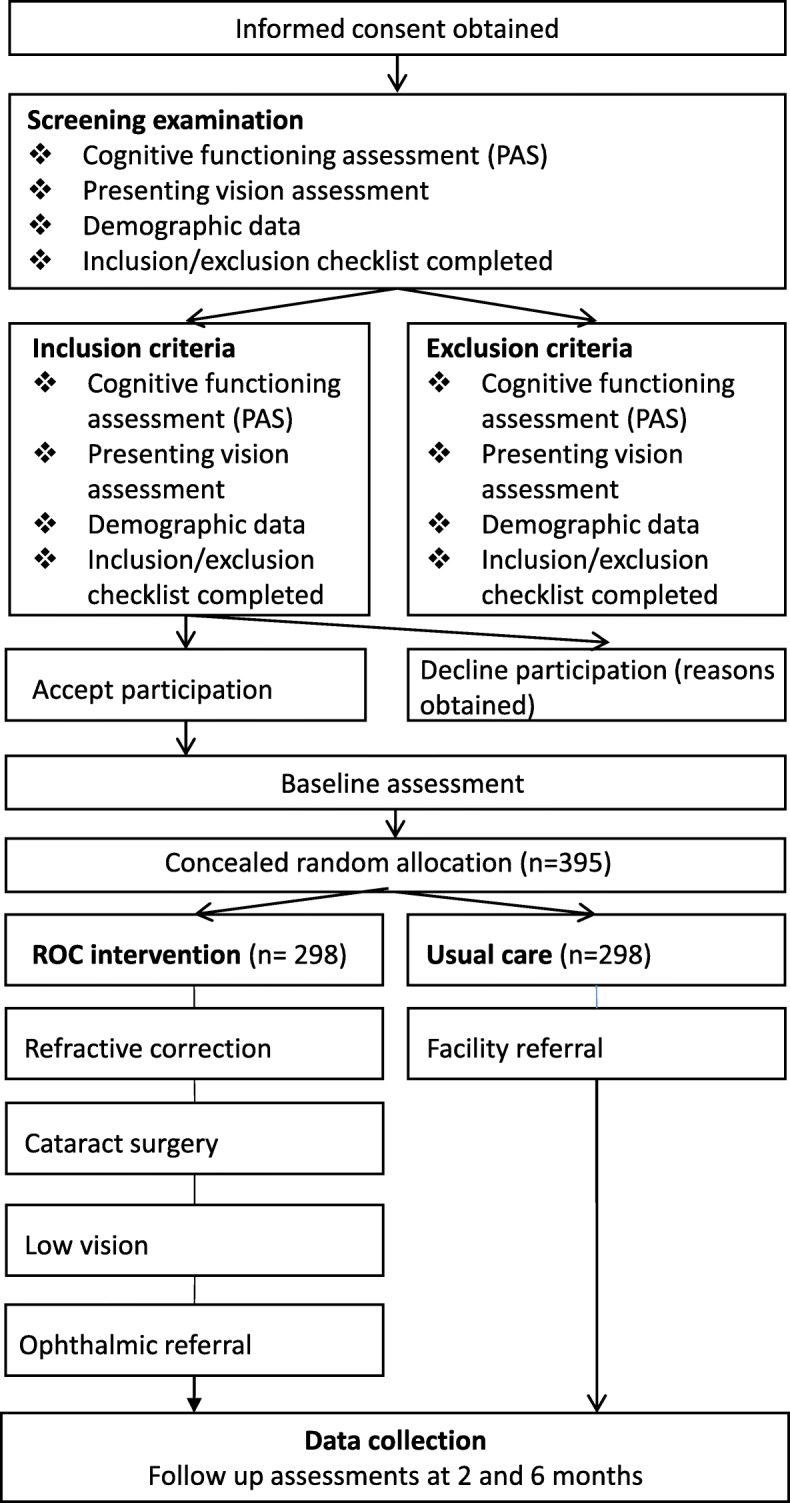
Participant flow and timeline for trial improving eye care in residential aged care facilities using the Residential Ocular Care (ROC) model

**Fig. 2:**
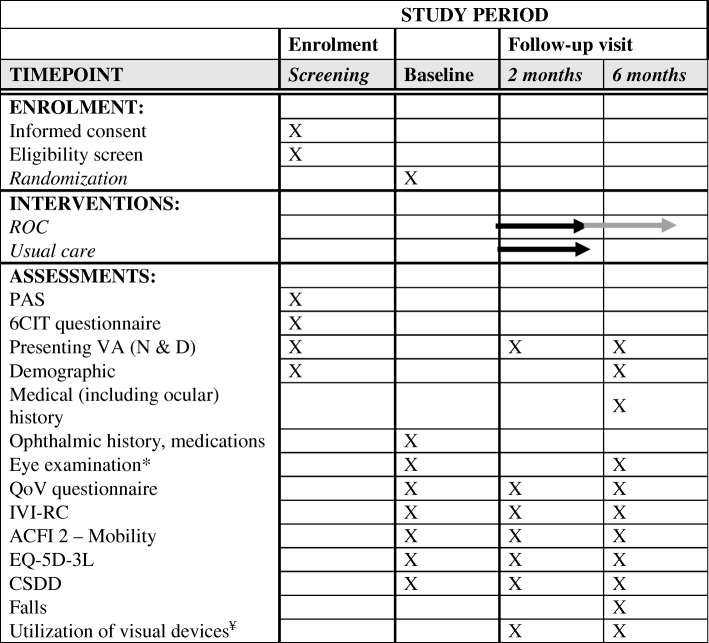
Schedule of enrolment, interventions, and assessments

A multicentered, prospective, and cluster RCT has been designed in accordance with the CONSORT statement and the Helsinki declaration. The study has been approved by the Royal Victorian Eye and Ear Hospital (RVEEH; reference number 15/1232H), Mercy Health (reference number R13-52 AC) and the Australian College of Optometry (ACO; reference number H14 001) Human Research Ethics Committees. This trial is subject to annual review and auditing by the RVEEH Human Research Ethics Committee. The trial has been registered with the Australian and New Zealand Clinical Trials Registry, number ACTRN12615000587505. The study design outlining the ROC intervention and standard treatment participant recruitment process is shown in Fig. 1. A Standard Protocol Items: Recommendations for Interventional Trials (SPIRIT) Checklist is provided as Additional file 1 and a flow diagram is included as Fig. 2.

### Settings and study participants

Prospective participants will be recruited from 38 aged care facilities in urban and rural Victoria, Australia. Before enrolment or study assessments being performed, prospective participants will be asked to sign a written consent form. Residential care staff will identify participants who are cognitively able to give informed consent. Participants who consent to participate in the study will be screened for study eligibility by a research assistant.

The inclusion criteria are:

1. Presenting distance < 6/12 (0.30; Logarithm of the Minimum Angle of Resolution-LogMAR) and/or near vision ≤ N8 (1.00 M) assessed uniocularly and binocularly using the Bailey-Lovie LogMAR [16] charts;
2. Able to speak English;
3. Moderate cognitive functioning or better as assessed by the Psychogeriatric Assessment Scale (PAS) [17] (score ≤ 15);
4. Ability to undergo VA testing and provide reliable results.

The exclusion criteria are:

1. Residents in respite care (short-term stay);
2. Severe cognitive impairment (score of ≥ 16 on the PAS);
3. Non-English speaking;
4. Inability to be tested for vision;
5. Contraindication(s) as indicated by the general practitioner responsible for the resident’s care.

### Randomization

An independent statistician will perform the randomization sequence generation using a computer-generated list. For logistical reasons and to minimize confounding effects, stratified cluster randomization designs will be used to randomize participants by residential aged care facility (stratified by size and region) to the intervention (ROC) or control (usual care) arm. Participants in the same aged care facility will all be assigned to the same study arm. The allocation sequence will be concealed from the research assistants enrolling and assessing participants. Residential care facility staff, the research study coordinator, and the eye health professionals delivering ROC will not be masked to the group allocation as this is impractical.

### Participant flow

All prospective participants who sign a study consent form will undergo a screening examination. When inclusion and exclusion criteria are met, the participant will be invited to complete a pre-intervention face-to-face baseline assessment with a research assistant. Following the baseline assessment, participants will receive the ROC intervention or usual care. All participants will be invited to complete follow-up assessments at two and six months after baseline visits.

### Intervention

The ROC model of eye care includes an on-site eye examination by an optometrist with expertise in domiciliary and low vision care. Four intervention options will be provided to help improve vision based on the individual participants’ eye history. These include: (1) refraction and spectacle provision; (2) cataract surgery; (3) referral to an ophthalmologist for medical and surgical treatments for conditions likely to cause loss of sight or ocular discomfort; and (4) low vision rehabilitation for untreatable eye disease. If a clinical need is identified, participants will be eligible to receive more than one intervention pathway (e.g. spectacles and low vision rehabilitation aids/services). For all pathways, transportation costs for initial consultations and for up to two follow-up consultations (to either a public or private care provider) will be funded by the study.

#### Refractive correction

An optometrist from the ACO will perform a detailed refraction for distance and near vision and measure best corrected VAs. Participants will be allocated to the refractive correction pathway if they meet the following criteria:

- Presenting VA < 6/12 (0.3 LogMAR) for distance or N8 (1.00 M) or worse for near which is improved by refractive correction by at least two lines/10 letters on a LogMAR Chart and/or 0.2 Log units for near in at least one eye;
- Participant has no other ocular co-morbidities requiring referral or urgent treatment that would affect the outcome of the refractive assessment and prescription of glasses.

If participants meet the above criteria, they will be prescribed, dispensed, and supplied with the appropriate spectacles from a selected range according to their visual needs and activities. One pair of spectacles (or two if separate distance and reading glasses are needed) will be provided and dispensed by the ACO and funded by the study.

#### Cataract surgery

The ACO optometrist will determine whether a participant will be referred for cataract surgery assessment by an ophthalmologist following the grading of lens opacities using the grading World Health Organization (WHO) Simplified Cataract Grading System [18]. Participants will be allocated to the cataract surgery pathway if they meet the following criteria:

- VA < 6/12 (0.3 LogMAR) that is not corrected by refractive correction by > 2 lines/10 letters;
- Phakic in at least one eye (can be pseudophakic in one eye);
- Evidence of lens opacities on anterior segment examination;
- No other ocular conditions identified by fundus or self-report requiring referral or urgent treatment before cataract surgery.

If spectacles are required following cataract surgery, these will be dispensed by ACO and funded by the study.

#### Referral to an ophthalmologist

A referral to an ophthalmologist will be provided if the participant has unexplained poor VA (< 6/12; 0.3 LogMAR) that is not due to uncorrected refractive error or cataract or shows evidence of AMD, DR, and/or glaucoma, and are not currently receiving ophthalmic advice/treatment for these conditions. Following medical consultation with a resident, treatment options, both surgical and medical, may be offered through the public health system, at the discretion of the treating ophthalmologist. Treatment options could include intraocular injections for conditions such as wet AMD and DR, surgical interventions for advanced glaucoma (i.e. trabeculoplasty), and the provision of topical medication for glaucoma, ocular inflammation, lid disease, and ocular infection. Other retinal eye conditions that require referral include (but are not limited to): retinal vein occlusion or emboli; macular hole; retinal detachments; retinal collaterals; and naevus. The study coordinator will liaise with the residential facility in organizing the initial ophthalmologist appointment and two subsequent follow-up appointments as required.

#### Low vision rehabilitation

Participants with VA < 6/12 (0.3 LogMAR) not correctable by either refraction or cataract surgery will be eligible for the low vision rehabilitation pathway. An ACO optometrist will undertake an initial comprehensive vision and ophthalmic review and provide low vision aids where appropriate at no cost. The type of low vision aids provided will be determined by the level of VI, the level of magnification required to perform desired tasks and the participant’s ability to use aids of different designs. Detailed demonstration, training, and instruction on aids will be given to the participant. Details of this examination will be forwarded with referral to the nearest Vision Australia center, Australia’s leading provider of blindness and low vision rehabilitation services, where an appointment will be scheduled within eight weeks. At this appointment, a Vision Australia Occupational Therapist will conduct a “Techniques for Daily Living” session with the participant, focusing on areas of difficulty and concern. Each session will be adapted to the individual circumstances of the resident, but be based around application of the following techniques:

- *Lighting:* general and/or task lighting;
- *Size:* bring things closer, make them larger, and vary the use of prescribed magnification;
- *Contrast:* increase the contrast between the foreground and the background;
- *Senses:* touch, scent, sound, and taste;
- *Marking:* tactile and/or contrast colored marking;
- *Labeling:* large print, Braille, or audio labels;
- *Organization:* different systems for organizing belongings;
- *Equipment:* everyday devices and/or functional use of low vision aids.

### Usual care

Residents with VI in the usual care group will be referred for an evaluation to the eye care service associated with the facility or a practitioner of their choice.

### Outcomes

#### Distance and near vision (primary)

Distance presenting VA at the two- and six-month follow-up visits after baseline. Participants’ uniocular and binocular distance VA (LogMAR charts) will be assessed, according to established protocols with their current presenting correction.

Uniocular and binocular presenting near VA will be assessed using LogMAR word reading cards, viewed at habitual working distances in the range of 25–40 cm. Reading fluency will be assessed as the time taken to read a block of text (Pepper Visual Skills for Reading Test cards) 0.3 log units larger than threshold. Following refraction, VAs at distance and near and contrast sensitivity will be assessed through best corrected VA.

For distance uniocular and binocular presenting VA, mild-moderate VI will be categorized as VA > 0.3 and ≤ 1.0 log units, while severe VI will be defined as VA > 1.0 log units. The proportion in each category will be compared between groups.

#### Other ocular outcomes (secondary)

Quality of vision will be measured using the 30-item Quality of Vision (QoV) scale [19]. Ten symptoms (e.g. glare, hazy vision, blurred vision, distortion) are rated on 4-point scales for Frequency (never, occasionally, quite often, very often), Severity (not at all, mild, moderate, severe), and Bothersome (not at all, a little, quite, very). The QoV results in three scales of frequency of visual symptoms, severity of symptoms, and how bothered the participant is by the symptoms. Higher mean scores represent greater Frequency, Severity, and Bothersomeness of visual symptoms.

Other aspects of visual function will be assessed (visual fields, color vision, and glare and contrast sensitivity [Pelli-Robson charts]) using instruments suitable for domiciliary assessment in accordance with clinical indications and protocols. Pathology will be graded using standardized grading scales. The integrity of the macular and optic disc will be further assessed by optical coherence tomography.

#### Person-centered outcomes (secondary)

Mobility will be assessed using the Aged Care Funding Instrument (ACFI) [20] module 2 developed by the Department of Health and Ageing. This component of the ACFI relates to the participant’s usual day-to-day assessed care needs with regard to mobility. Mean mobility scores will be compared between groups. ACFI data will be collected regularly by residential care facility staff.

Vision-specific QoL will be measured using the Impact of Vision Impairment for Residential Care (IVI-RC). The IVI-RC is a 28-item questionnaire developed to measure the impact of vision impairment on activity limitation and emotional wellbeing, and has been adapted specifically to the aged care setting by the study team from the original 28-item IVI questionnaire [21, 22].

Depressive symptoms will be determined using the Cornell Scale for Depression in Dementia (CSDD) [23]. This measure was chosen due to its suitability for use with individuals with moderate cognitive functioning. The interviews focus on depressive symptoms and signs occurring during the week before the interview. The final ratings of the CSDD represent the rater’s clinical impression. Each item is rated for severity on a scale of 0–2 (0 = absent, 1 = mild or intermittent, 2 = severe). The item scores are added together. Scores of 0–8, 9–13, 14–18, and 19–38 indicate no or minimal, mild, moderate, and major depressive symptoms [24].

Regarding falls rate, residential care settings have an incident reporting system (which varies from paper based to computerized systems) where any incident including falls will be recorded. Falls data will be retrieved from incident reporting systems in the participating residential care facilities and the proportion of falls will be compared between groups.

Health-related QoL will be assessed using the five-dimension EuroQoL (EQ-5D-3L) [25], a descriptive system that covers five dimensions (Mobility, Self-care, Usual Activities, Pain/Discomfort, and Anxiety/Depression). The EQ-5D-3L raw data can be converted to utilities, which will be used for our economic analyses.

With regard to utilization of visual devices (distance and near vision glasses; low vision aids), at the two- and six-month follow-up visits, participants in the intervention arm who have been prescribed spectacles or low vision aids, will be asked to self-report on how often the device has been used during the week, for how long it was used, and for what tasks.

All QoL outcome measures will be interviewer administered. The IVI-RC and the CSDD will initially undergo Rasch Analysis (a form of Item Response Theory) using Winsteps software (version 3.92.1; Chicago, IL, USA) [26] in order to convert the ordinal questionnaire scores to estimate of interval level measures. Mean Rasch scores for the IVI-RC and CSDD will be compared between groups.

### Retention and withdrawal

We will seek to follow up all participants except those who expressly withdraw from the study. Withdrawal at the participant level will be considered if any of the following occurs: participant is lost to follow-up; withdrawal of consent; or death. All withdrawals will be recorded and the reason will be specified.

### Adverse events

Only study-related adverse events (AE) will be reported. Information regarding AEs (including incidence, duration, seriousness, severity, relationship to treatment, and action taken) will be recorded throughout the study. If AEs occur, the first concern will be the safety of the study participants. AEs will be graded by the research staff completing the assessments and/or residential facility physicians at each site for severity and relationship to study treatment. Study-related AEs will also be reported to the site’s Human Research Ethics Committee.

### Statistical analysis

The primary and secondary outcomes will be assessed using an intention-to-treat analysis applied to individual participants and repeated using the per-protocol sample (only participants who complete the clinical trial including the second assessment). To compare the effectiveness of ROC with usual care, presenting and best corrected near and distance VA (primary outcome) at the two- and six-month follow-ups will be analyzed and the differences from baseline VA will be used to investigate changes. The near and distance VA change is expected to be normally distributed and analysis of covariance will be used to compare the difference, adjusting for baseline values. Effect sizes for both groups will be calculated using Cohen’s d coefficient [27]. To determine the effectiveness of ROC on secondary outcomes, a Multivariable General Linear Model will be used to compare differences at two and six months from baseline on the IVI-RC, EQ-5D-3L and CSDD. If the EQ-5D-3L data are skewed, a multivariable quantile regression model will be used. Falls data will be compared across intervention and usual care groups at six months using a negative binomial regression if the data are over-dispersed. Changes in mobility will be analyzed using Mann–Whitney U tests. The difference in utilization of distance and near vision glasses and low vision aids between the treatment groups will be compared using the Chi-squared test and the utilization time will be compared using Student’s t-test or non-parametric test if appropriate. A multi-level model with a random intercept parameter will be utilized to account for within cluster correlations. All analyses will be performed using STATA version 15 (StataCorp. 2017. Stata Statistical Software: Release 15. StataCorp LLC, College Station, TX, USA).

### Sample size

Pilot data collected from 31 participants residing in a single-site aged care facility were used to guide power calculations for this study (Constantinou M, Nicolaou T, Jackson J, Lamoureux E: Vision Optimisation in Residential Care: A pilot study, unpublished, 2011). They showed that following correction, 30% of participants had improved distance vision by at least two lines and 30% of the participants improved by ≥ 0.2 Log units on a text reading chart for near vision. Therefore, the sample size calculation is based on achieving a 30% improvement in VA (near and distance vision) at two- and six-month follow-up visits in the intervention group compared to controls. The anticipated effect size will be ~ 0.65 based on our pilot work. At a significance level of 0.05, with 38 participants in each arm (overall total of 76 participants) we will have 80% power to detect a group difference. We assume an intra-cluster correlation coefficient (ICC) within-facility of 0.05 which corresponds to the levels found in similar work in nursing homes [28]. The inflation factor (or design effect) will be estimated using the following formula: Inflation factor = 1+ (number of patients per facility [50]-1) X ICC = 3.5. To take cluster design into account, we need a total sample size of 266 [76 × 3.5]. However, to adjust for non-compliance with the intervention (~ 10% due to people who seek vision care on their own in the usual care arm, or people who refuse the intervention in the intervention arm) and loss to follow-up (~ 25% largely due to deaths), the effective sample size needs to be increased by a factor of 1.48 (1/0.90 × 1/0.75) resulting in an initial enrolment requirement of 395 individuals.

### Missing data

The differences between the intervention and usual care groups in the proportion and reason of participant withdrawal will be compared using the Chi-squared test. The multiple imputations method using chained equations (MICE) will be used to handle missing data.

### Cost-effectiveness analysis

We will perform an incremental cost-effectiveness analysis from the payer perspective. Direct intervention costs (labor, material and supplies, and contracted services costs) associated with delivering the intervention over the one-year period will be collected and follow a cost collection approach described elsewhere [29]. The incremental costs of the intervention will need to account for any vision services received by the usual care group and any differences in costs for non-vision related services that may be an indirect result of the intervention. Information will be collected for any participants who fall or are hospitalized, transferred to high care or die. Aggregating these costs over the six-month period and adding them to the direct costs of the ROC intervention group, will allow the total medical costs for the intervention and the usual group to be quantified. The incremental cost-effectiveness ratio (ICER) will be calculated using the estimated mean difference in effect (EQ-5D-3L) and the estimated mean difference in costs between the intervention and the control groups. The ICER indicates the average incremental cost to gain one additional quality-adjusted life year (QALY). Cost data are likely to be skewed. Therefore, using non-parametric bootstrapping, 1000 replicates [30] will be generated to estimate the 95% confidence intervals around the ICER. A cost-effectiveness acceptability curve (CEAC) [31] will be used to show the probability that the intervention is cost-effective for a range of monetary values that a decision maker might be willing to pay for a unit change in QALYs. Cost-effectiveness will initially be quantified assuming that QALYs are only sustained for one year, but an assessment will also be made based on the assumption that they continue to accrue each year for the expected remaining life years in the study sample. Additional one-way (and n-way) deterministic sensitivity analyses will be performed to examine the effect of changing one (or n) of the model parameters.

### Ethics and dissemination

The study protocol (Version 2.2, 10 May 2016) was reviewed and approved by the RVEEH (reference number 15/1232H), Mercy Health (reference number R13-52 AC), and the ACO (reference Number H14 001) Human Research Ethics Committees. Any protocol modifications will be sent for review by the research ethics committee and will be amended at the trial registry (Australian and New Zealand Clinical Trials Registry, number ACTRN12615000587505). The principal investigator is responsible for informing the research ethics committee and trial registry of any amendments to the protocol or other study-related documents.

A unique ID will be assigned to all participant who are successfully enrolled on the study. Study questionnaires will only refer to the participants using this ID number. In addition, data will be de-identified before is it passed to the statistician (XJ). Records containing identifiable data, such as the Consent Form, will be stored in locked cabinets at the Centre for Eye Research Australia with restricted access. Only the investigators and authorized personnel directly involved with the study will have access to the data. All data files will be password protected and stored on a secure server at the Centre for Eye Research Australia for 10 years and then securely destroyed.

It is planned that results will be disseminated to academic and health professional audiences via presentations at conferences and publication in peer-reviewed journals. Participants will be sent a summary of the trial findings at the time when the main article is published. The trial results will be communicated to policymakers through briefing papers summarizing the main findings. We will also provide the results to all participants and disseminate the results to the public through a press release, regardless of what the results show.

## Discussion

This study protocol is the first multicentered, customized, prospective, and cluster RCT to examine the effect of a novel eye care model on VA as well as person-centered outcomes for aged care residents in Australia. Previous trials in this setting have found short-term benefits for single treatment pathways, indicating that refractive error correction or cataract surgery could enhance functional status and vision-targeted health-related QoL, in addition to improving vision [11, 32, 33]. However, our trial will be the first to evaluate a comprehensive, person-centered, and customized eye care model that includes refraction and spectacle provision, cataract surgery, low vision rehabilitation, and/or a referral to an ophthalmologist for medical and surgical treatments for conditions likely to cause loss of sight or ocular discomfort.

A major strength of this trial is the provision and integration of a range of eye care services for a broad spectrum of ophthalmic conditions, including those which cannot be treated through refractive correction or cataract surgery, the inclusion of both urban and regional facilities, and the investigation of clinical optometric as well as QoL and psychological outcomes. The findings from this trial will also yield novel data on longer-term effectiveness of eye care services delivered to this vulnerable population. The ROC model described in this protocol has been designed to be feasible, cost-effective, and easy to implement and potentially delivered in any residential care setting, which may have direct clinical applications.

Australia, similar to other developed countries, is facing unprecedented challenges to meet the growing healthcare needs of an aging population. It is predicted that by 2050, upwards of 3.5 million Australians will be accessing nursing and residential aged care services annually. This growth will occur alongside a forecasted health workforce shortage and a decreasing number of primary care and specialist health providers visiting facilities [34, 35]. In an effort to meet this increased demand, different healthcare service delivery models are needed. Our innovative intervention that targets both treatable and non-treatable causes of VI is likely to have a substantial beneficial effect in improving eye health, participation in daily living activities, falls, emotional wellbeing, and QoL. The tailored intervention will potentially empower older adults living in facilities to remain to a greater extent independent and in charge of their own lives, a key focus of both national and international frameworks to promote healthy aging [36, 37].

More broadly, the challenges for evaluating clinical and healthcare in residential aged care are many. Delivering effective care should be a priority for aged care providers given the high burden of chronic disease and comorbidity. An informative starting point could be to target the management of the most prevalent conditions and co-morbidities. Our ROC model will provide a new approach to optimize the synergies of groups working with the elderly in the residential care environment, leading to cost-effective and sustainable approaches to minimize VI and associated declines in functioning and QoL. It is hoped that the combined ophthalmological, optometric, and rehabilitation approach, reflected in the ROC study design, will further assist health and social care planners in developing new and innovative models of care for this often-overlooked population sub-group.

The ROC study is the first multicentered, prospective, customized, and cluster RCT in Australia to determine the effectiveness of a comprehensive and tailored eye care model for people residing in aged care facilities. This study will evaluate intervention outcomes with respect to improvements in visual acuity; as well as person-centered outcomes. We will also conduct a cost-effectiveness analysis to quantify the health improvements gained relative to the resources expended. Our study will provide evidence to determine whether this customized model is an appropriate strategy for implementing in residential care settings both in Australia and elsewhere.

### Trial status

The present publication refers to the ROC study protocol version 2.2, 10 May 2016. Recruitment began on 18 June 2014 and is expected to be completed by 1 December 2018.

## Additional file

Additional file 1: SPIRIT 2013 Checklist: Recommended items to address in a clinical trial protocol and related documents*. (DOC 120 kb)

## Abbreviations

ACFI: Aged Care Funding Instrument
ACO: Australian College of Optometry
AE: Adverse events
AMD: Age-related macular degeneration
CEAC: Cost-effectiveness acceptability curve
CSDD: Cornell Scale for Depression in Dementia
DR: Diabetic retinopathy
EQ-5D-3L: 3-level Euro-Qol
ICC: Intra-cluster correlation coefficient
ICER: Incremental cost-effectiveness ratio
IVI-RC: Impact of Vision Impairment for Residential Care
LogMar: Logarithm of the Minimum Angle of Resolution
MICE: Multiple imputations by chained equations
PAS: Psychogeriatric Assessment Scale
QALYs: Quality-adjusted life years
QoL: Quality of life
QoV: Quality of Vision Scale
RCT: Randomized controlled trial
ROC: Residential Ocular Care
RVEEH: Royal Victorian Eye and Ear Hospital
VA: Visual acuity
VI: Vision impairment
WHO: World Health Organization
